# Application of Community Detection Methods to Identify Emergency General Surgery–Specific Regional Networks

**DOI:** 10.1001/jamanetworkopen.2024.39509

**Published:** 2024-10-15

**Authors:** Jiuying Han, Neng Wan, Joshua J. Horns, Marta L. McCrum

**Affiliations:** 1 Department of Geography, University of Utah, Salt Lake City; 2 Surgical Population Analysis Research Core, Department of Surgery, University of Utah, Salt Lake City

## Abstract

**Importance:**

There is growing interest in developing coordinated regional systems for nontraumatic surgical emergencies; however, our understanding of existing emergency general surgery (EGS) care communities is limited.

**Objective:**

To apply network analysis methods to delineate EGS care regions and compare the performance of this method with the Dartmouth Health Referral Regions (HRRs).

**Design, Setting, and Participants:**

This cross-sectional study was conducted using the 2019 California and New York state emergency department and inpatient databases. Eligible participants included all adult patients with a nonelective admission for common EGS conditions. Interhospital transfers (IHTs) were identified by transfer indicators or temporally adjacent hospitalizations at 2 different facilities. Data analysis was conducted from January to May 2024.

**Exposure:**

Admission for primary EGS diagnosis.

**Main Outcomes and Measures:**

Regional EGS networks (RENs) were delineated by modularity optimization (MO), a community detection method, and compared with the plurality-based Dartmouth HRRs. Geographic boundaries were compared through visualization of patient flows and associated health care regions. Spatial accuracy of the 2 methods was compared using 6 common network analysis measures: localization index (LI), market share index (MSI), net patient flow, connectivity, compactness, and modularity.

**Results:**

A total of 1 244 868 participants (median [IQR] age, 55 [37-70 years]; 776 725 male [62.40%]) were admitted with a primary EGS diagnosis. In New York, there were 405 493 EGS encounters with 3212 IHTs (0.79%), and 9 RENs were detected using MO compared with 10 Dartmouth HRRs. In California, there were 839 375 encounters with 10 037 IHTs (1.20%), and 14 RENs were detected compared with 24 HRRs. The greatest discrepancy between REN and HRR boundaries was in rural regions where one REN often encompassed multiple HRRs. The MO method was significantly better than HRRs in identifying care networks that accurately captured patients living within the geographic region as indicated by the LI and MSI for New York (mean [SD] LI, 0.86 [1.00] for REN vs 0.74 [1.00] for HRR; mean [SD] MSI, 0.16 [0.13] for REN vs 0.32 [0.21] for HRR) and California (mean [SD] LI, 0.83 [1.00] for REN vs 0.74 [1.00] for HRR; mean [SD] MSI, 0.19 [0.14] for REN vs 0.39 [0.43] for HRR). Nearly 27% of New York hospitals (37 of 139 hospitals [26.62%]) and 15% of California hospitals (48 of 336 hospitals [14.29%]) were reclassified into a different community with the MO method.

**Conclusions and Relevance:**

Development of optimal health delivery systems for EGS patients will require knowledge of care patterns specific to this population. The findings of this cross-sectional study suggest that network science methods, such as MO, offer opportunities to identify empirical EGS care regions that outperform HRRs and can be applied in the development of coordinated regional systems of care.

## Key Points

**Question:**

How does detection of emergency general surgery (EGS) care communities using network analysis methods compare with the Dartmouth Health Referral Regions (HRR) with respect to geographic boundaries and network accuracy?

**Findings:**

In this cross-sectional study of 1 244 868 patients receiving EGS in New York and California, EGS care regions detected by the community detection method were distinct from those generated by the Dartmouth HRR method, with superior localization of patients to individual communities.

**Meaning:**

The findings suggest that EGS care regions delineated by community detection methods are reliable and more specific than alternate methods using the general Medicare population.

## Teaser

This cross-sectional study uses network analysis methods to identify emergency general surgery (EGS) care regions in New York and California and compares this method of identification with Dartmouth health referral regions.

## Introduction

Emergency general surgery (EGS) conditions represent a substantial public health burden, with more than 3 million admissions annually in the US, and mortality, morbidity, and health care utilization that far exceed similar elective conditions.^1,2,3^ Consequently, national organizations including the American College of Surgeons (ACS) and the American Association for the Surgery of Trauma (AAST) have acknowledged the need to improve health systems for this high-risk and complex patient population, and have introduced the EGS hospital verification program as a first step in formalizing care for nontraumatic surgical emergencies.^4^

Regionalized care systems have been implemented for other time-sensitive conditions, including trauma, stroke, neonatal intensive care unit care, and ST-elevation myocardial infarction by standardizing care delivery processes and matching patients with hospitals best able to meet their clinical needs.^5,6,7,8,9^ For these conditions, regionalized systems have been shown to improve the quality of care and time to intervention, and to reduce mortality.^10,11,12^ Due to the time-sensitive nature of EGS conditions and wide national variation in quality and outcomes, a regionalized approach has been proposed for nontraumatic surgical emergencies as well.^13,14,15^ Doing so, however, will require a detailed understanding of the existing landscape of EGS care, including utilization patterns within geographic regions and interhospital transfer (IHT) patterns.

Network analysis methods offer new opportunities to analyze patterns of care specific to EGS disease. To date, most studies of surgical health service utilization, regional performance, and health disparities have used the Dartmouth hospital referral regions (HRRs) to define regions of care.^16^ HRRs were defined using cardiovascular and neurosurgical referral patterns and a plurality-based approach, where zip codes were associated with towns or cities containing the hospitals where residents receive the most care, with adjacent regions subsequently aggregated.^17^ While widely used, there are numerous concerns regarding the effectiveness of the Dartmouth HRRs.^18^ Chief among them is that HRRs were derived using 1992 to 1993 Medicare data that are now more than 30 years old and not reflective of current hospitalization patterns due to changes in health care infrastructure (eg, hospital closures, openings, or mergers), population changes (eg, demographic and geographic shifts), and insurance market changes. Moreover, the general Medicare population is unlikely to be representative of the overall adult population seeking emergency care for underlying surgical disease.

An alternative to use of HRRs is to apply community detection methods to current data. One such method is modularity optimization (MO), a data-driven optimization technique of delineating communities by maximizing within-region flows while minimizing between-region flows.^19,20,21^ This approach has been applied widely in social network analysis, with growing traction in health care.^22,23^ In this study, we sought to apply MO specifically to patients admitted with EGS conditions to identify existing networks of EGS care and compare the performance of this method with the Dartmouth HRRs.

## Methods

### Data Sources and Patient Population

This cross-sectional study was determined to be exempt from review and the requirement of informed consent by the University of Utah institutional review board because it was determined not to be human participants research and used deidentified data. The study followed the Strengthening the Reporting of Observational Studies in Epidemiology (STROBE) reporting guideline. We used the 2019 Healthcare Cost and Utilization Project (HCUP) state emergency department and state inpatient databases for California and New York.^24,25^ We selected California and New York due to their large geographic size, with considerable rural and urban areas and adjacency to a water body, leading to decreased interstate patient flows. We identified all adult patients (≥18 years), with a nonelective presentation and primary discharge diagnosis of 1 of 12 common EGS conditions: appendicitis, cholecystitis, diverticulitis, small bowel obstruction, infectious colitis, esophageal perforation, peptic ulcer disease, mesenteric ischemia, pancreatitis, perirectal abscess, hernia, or soft tissue infection using *International Statistical Classification of Diseases, Tenth Revision, Clinical Modification (ICD-10-CM) *codes (eTable in Supplement 1).^26,27^ These represent the most common EGS diseases identified by the AAST and form the core scope of the ACS EGS verification program.^28^ IHTs were identified by transfer indicators or temporally adjacent hospitalizations (ie, the discharge date from the first hospital and the admitting date to the second hospital are within 0-1 day) at 2 different facilities using the visitlink variable to follow patients longitudinally and across the state inpatient and state emergency department datasets.^29^ Patients were excluded if site of admission was from a location other than home. Hospital characteristics, including hospital location, inpatient surgical volume, and trauma center designation (with level 1 and 2 representing hospitals with advanced clinical resources), were obtained from the 2020 American Hospital Association annual survey.

### Detection of EGS Care Network Structure

We used the Louvain community detection method, an algorithm developed using the MO theory, to detect the existing EGS care network structures.^30^ To differentiate them from the Dartmouth HRRs, we named these communities the regional EGS networks (RENs).

In network analysis, the Louvain algorithm is often used to group nodes into communities by maximizing a measure named modularity to find the best division of the nodes in the network. Modularity compares the number of edges within a community to the number of edges within a random network. Higher modularity scores, indicating that the edges are more densely connected than a random network, suggest better community detection. The modularity of a weighted network is calculated as:
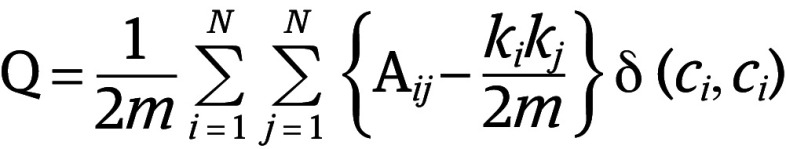
,where *A_ij_* represents the edge weight between node *i* and *j, k_i_* and *k_j_* are the sum of weights connected to nodes *i* and *j,* respectively, *m* is the sum of all the weights in the network, *N* is the number of nodes in the network, *c_i_* and *c_j_* are the communities of nodes *i* and *j*; σ is equal to 1 if *c_i_* and *c_j_* are within the same community; σ is 0 otherwise.^31^

The steps of community detection are as follows. In the first step, all the nodes are treated as separate communities. In the second step, neighboring communities are combined, and the modularity difference is calculated. The communities stay combined if there is a modularity gain. Then, the first and the second steps are repeated based on newly formed communities until no modularity gain can be achieved.

In this study, we conducted 2 phases of REN detection. The overall EGS care network (phase 1) represents patient flow from home to the initial hospital. The IHT network (phase 2) captures patient flows between hospitals for those who underwent transfer. The 2 phases combined represent the 2 most important processes of EGS care delivery and thus were used to reveal the structures of regional EGS care networks. Because the geographic identifier available in the HCUP datasets is the residential zip code, zip code tabulation areas were used for the geographic unit of aggregation. In phase 1, patients (represented by their residential zip code centroids) and hospitals were abstracted into nodes. The process of patients being admitted to hospitals connects the patients to their admitted hospitals by an edge to form a network. The edge weights of the network are patient volumes from the same zip code centroids to the same hospitals. The communities of the original EGS care network were detected using the previously described steps. In phase 2, the network is built upon phase 1. The communities detected in phase 1 become the nodes for the second phase. The process of IHT connects the phase 1 communities to form a network. In this phase, the edge weights are the patient volumes transferring from the same sending communities to the same receiving communities. The final communities of the EGS IHT (phase 2) network are the RENs. The analysis was conducted using the igraph package in R version 4.3.1 (R Project for Statistical Computing).^32^

### Visualization

To visually compare the 2 methods, we overlaid the Dartmouth HRRs on top of the MO RENs. We added layers of hospitals and IHT flows to show physical relationship of the EGS care networks with the detected communities. Visualization of hospitals included inpatient surgical volume and trauma center designation to show the hierarchy of the hospitals within the EGS care networks. Force-directed edge bundling was used to visualize IHT flows to reduce visual clutter and to reveal the patterns of the flows.^33^ The visualization was performed with R leaflet package and force-directed edge bundling with the R package edgebundle.^34,35^

### Statistical Analysis

We compared the spatial accuracy of the MO RENs with Dartmouth HRRs in identifying EGS communities using common network analysis measures that have been applied to health care networks.^36,37^ Six metrics, localization index (LI), market share index (MSI), net patient flow (NPF), connectivity, compactness, and modularity were used for the evaluation (Table 1).^18,21,31,38^ LI reflects the proportion of patients that are treated in the same community as where they live, MSI reflects the proportion of patients treated in a given community who live in another community, and the NPF is the ratio of incoming patients to outgoing patients of a community. An ideally detected community would have high LI, low MSI, and an NPF close to 1. Due to the small number of communities, the Mann-Whitney *U* test was used to assess significance, with significance assigned at a 2-sided *P* < .05. Additionally, we also compared the community classification differences between the 2 methods by examining the percentage of hospitals in each HRR reclassified to a new REN network using the MO method, using the HRR with the corresponding central transfer hub as a reference point. Data analysis was conducted from January to May 2024.

**Table 1.** Spatial Accuracy and Network Characteristics Measures

Measure	Definition
Spatial accuracy	
Localization index	The proportion of patients that are treated in the same HRR or REN as where they live. A higher localization index means more accurate delineation of HRRs and RENs.
Market share index	The proportion of patients treated in a given HRR or REN who live in another region. A smaller market share index means better delineation.
Net patient flow	The ratio of incoming patients to outgoing patients of an HRR or REN. A net patient flow value closer to 1 means a more balanced HRR or REN structure.
Network characteristics	
Connectivity	Number of patient transfers among hospitals within an HRR or REN. A larger connectivity means that hospitals within the HRR are more connected.
Compactness	The regularity of a region’s shape based on the perimeter-area corrected ratio. A smaller compactness means the more compact a region is.
Modularity	Number of connections in the whole network compared with a completely random model. A larger modularity means better delineation.
Reclassification	
Hospital	The mean percentage of hospitals in each Dartmouth HRR that have been classified into different MO RENs.
Encounter: index hospital visit	The mean percentage of index hospital visits in each Dartmouth HRR that have been classified into different MO RENs.
Encounter: interhospital transfer	The mean percentage of interhospital transfer in each Dartmouth HRR that have been classified into different MO RENs

Abbreviations: HRR, health referral region; MO, modularity optimization; REN, regional emergency general surgery network.

## Results

In New York, there were 405 493 EGS encounters with 3212 IHTs (0.79%), and detected 9 RENs using MO, compared with 10 Dartmouth HRRs. In California, there were 839 375 encounters and 10 037 IHTs (1.20%) and detected 14 RENs vs 24 HRRs. Brief population demographics of the 1 244 868 participants (median [IQR] age, 55 [37-70] years; 776 725 male [62.40%]) are provided in Table 2.

**Table 2.** Study Population

Characteristic	Participants, No. (%)		
	New York (n = 405 493)	California (n = 839 375)	Total cohort (N = 1 244 868)
Age, median (IQR), y	56 (37-71)	55 (37-69)	55 (37-70)
Sex			
Female	199 589 (49.22)	393 042 (46.82)	592 631 (47.60)
Male	205 904 (50.77)	446 333 (53.17)	776 725 (62.40)
Race and ethnicity			
Asian or Pacific Islander	10 055 (2.48)	43 689 (5.20)	53 744 (4.32)
Black	62 971 (15.53)	78 019 (9.29)	140 990 (11.33)
Hispanic	58 450 (14.41)	260 475 (31.03)	318 925 (25.62)
Native American	1156 (0.29)	4608 (0.55)	5764 (0.46%)
White	243 803 (60.12)	419 984 (50.03)	663 787 (53.32)
Other^a^	29 058 (7.17)	32 600 (3.88)	61 658 (4.95)
Location of treatment			
Emergency department only	175 939 (43.39)	436 066 (52.0)	612 005 (49.20)
Inpatient admission	226 342 (55.80)	393 272 (46.95)	619 614 (49.77)
Interhospital transfer	3212 (0.79)	10 037 (1.20)	13 249 (1.06)
Primary emergency general surgery condition			
Appendicitis	21 430 (5.28)	29 179 (3.48)	50 609 (4.07)
Cholecystitis	19 727 (4.86)	37 752 (4.50)	57 479 (4.62)
Diverticulitis	24 426 (6.02)	45 864 (5.46)	70 290 (5.65)
Esophageal perforation	6251 (1.54)	17 491 (2.08)	23 742 (1.91)
Perforated ulcer	1254 (0.31)	2366 (0.28)	3620 (0.29)
Intestinal obstruction	7637 (1.88)	15 000 (1.79)	22 637 (1.82)
Infectious colitis	15 729 (3.88)	27 119 (3.23)	42 848 (3.44)
Mesenteric ischemia	5814 (1.43)	10 271 (1.22)	16 085 (1.29)
Pancreatitis	25 063 (6.18)	57 003 (6.79)	82 066 (6.59)
Perirectal abscess	5350 (1.32)	10 321 (1.23)	15 671 (1.26)
Hernia	93 181 (22.93)	168 214 (20.04)	261 395 (21.00)
Soft tissue infection	196 810 (48.54)	456 341 (54.34)	653 151 (52.47)

^a^ Other was defined as any race or ethnicity other than the categories reported.

### Visualization of the Communities

The Figure illustrates RENs and HRRs with overlaid IHT patient flows. While the overall distribution of RENs was similar to the distribution of HRRs, there were notable differences. The greatest discrepancy between REN and HRR boundaries was evident in rural regions where one REN may encompass multiple HRRs, such as the northeastern part of New York and northern California. Both methods had edges crossing community boundaries, although HRRs exhibited a higher frequency of boundary-crossing edges. For instance, in Otsego County in New York, several edges with large IHT flow volumes intersected the boundaries of HRRs, indicating that HRRs partition closely interconnected areas into different EGS care communities. A comparable situation unfolded in California, where the northern part was fragmented into numerous small HRRs, resulting in a great number of IHT flows traversing HRR boundaries.

**Figure.**
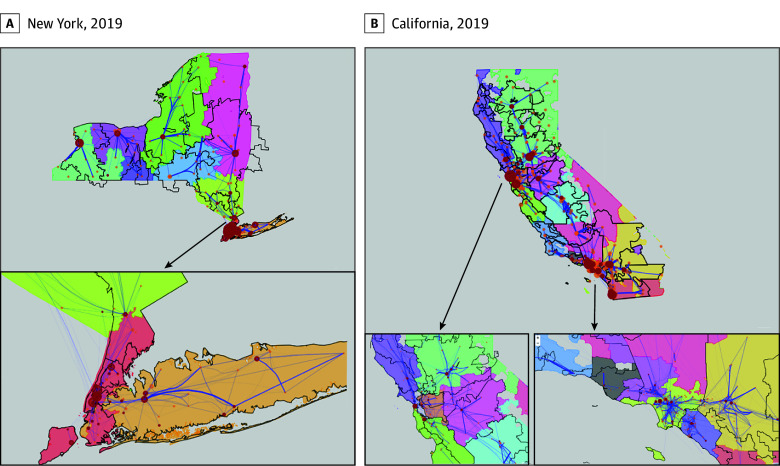
Distribution of Modularity Optimization–Derived Regional Emergency General Surgery (EGS) Networks and Dartmouth Health Referral Regions (HRRs) Regions with black boundaries indicate Dartmouth HRRs, while the various colored regions indicate modularity optimization Regional EGS Networks (RENS). Circles indicate hospitals, with red circles indicating a level 1 or 2 trauma center and orange circles indicating non–level 1 or 2 trauma centers. Larger circles denote higher volumes. The blue lines of varying thicknesses represent interhospital transfer flows between hospitals. In an optimally detected community, all interhospital transfer flows would remain within a region’s boundaries.

### Spatial Accuracy Metrics

Spatial accuracy metrics showed the MO method to be superior to the Dartmouth method in all the metrics except compactness (Table 3). The modularity of the RENs was much larger than HRRs in both New York (modularity, 0.69 for REN vs 0.63 for HRR) and California (mean [SD] modularity, 0.74 for REN vs 0.69 for HRR). The MO method was better than the Dartmouth method at identifying care networks that accurately captured patients living within the geographic region for both New York (mean [SD] LI, 0.86 [1.00] for REN vs 0.74 [1.00] for HRR; mean [SD] MSI, 0.16 [0.13] for REN vs 0.32 [0.21] for HRR) and California (mean [SD] LI, 0.83 [1.00] for REN vs 0.74 [1.00] for HRR; mean [SD] MSI, 0.19 [0.14] for REN vs 0.39 [0.43] for HRR). This finding can also be demonstrated through the visualization because there were a greater number of edges crossing HRR boundaries (Figure). In California, hospitals within RENs were more tightly connected to each other than HRR communities (connectivity). Using the MO method to detect regional communities resulted in reclassification of 37 of 139 hospitals in New York (26.62%) and 48 of 336 hospitals in California (14.29%) compared with the Dartmouth HRRs.

**Table 3.** Network Accuracy and Network Characteristics

Measure	New York			California		
	Modularity optimization–regional EGS network, mean (SD) [range] (n = 9)	Dartmouth health referral region, mean (SD) [range] (n = 10)	*P* value	Modularity optimization–regional EGS network, mean (SD) [range] (n = 14)	Dartmouth health referral region, mean (SD) [range] (n = 24)	*P* value
Spatial accuracy measures						
Localization index	0.86 (1.00) [1.00-1.00]	0.74 (1.00) [0.50-1.00]	<.001	0.83 (1.00) [0.75-1.00]	0.74 (1.00) [0.50-1.00]	<.001
Market share index	0.16 (0.13) [0.07-0.20]	0.32 (0.21) [0.16-0.41]	.07	0.19 (0.14) [0.11-0.24]	0.39 (0.43) [0.19-0.50]	<.001
New patient flow	2.14 (1.00) [0.20-1.93]	2.61 (0.62) [0.29-3.65]	.97	1.76 (1.25) [0.62-2.64]	1.44 (1.34) [0.35-1.82]	.39
Network characteristics						
Connectivity	191.6 (201.0) [152.0-235.0]	141.8 (136.5) [42.8-241.8]	.49	430.1 (282.5) [264.5-477.5]	215.6 (108.0) [46.0-208.0]	<.001
Compactness	3.79 (2.43) [2.41-3.05]	3.49 (2.93) [2.86-3.27]	.33	3.01 (2.73) [2.48-3.18]	2.79 (2.49) [2.27-3.03]	.27
Modularity, No.	0.69	0.63	NA	0.74	0.69	NA
Reclassification						
Hospital, median (IQR), %	14.67 (0.00-28.85)	NA	NA	9.90 (0.00-15.40)	NA	NA
Encounter						
Index hospital visit, median (IQR), %	10.12 (0.00-18.16)	NA	NA	8.57 (0.00-20.08)	NA	NA
Interhospital transfer, median (IQR), %	6.91 (0.00-9.85)	NA	NA	6.05 (0.00-10.44)	NA	NA

Abbreviations: EGS, emergency general surgery; NA, not applicable.

## Discussion

In this cross-sectional study, we demonstrate the application of MO, a data-driven and automated technique, to delineate the structure of RENs in 2 large, geographically diverse states. Compared with the long-established Dartmouth HRRs, MO-generated RENs more accurately assigned patients to care regions, and in California, identified hospital communities with stronger connections. Moreover, more than 10% of hospitals were reclassified into a new community with the MO-based method. These findings hold important implications for the development of regionalized systems for EGS care.

Over the past 3 decades, the Dartmouth Atlas Health Referral Regions have been widely used as the default geographic unit for surgical health services research and investigation into regional variation, disparities, value, and access to care for a wide range of surgical diseases.^39,40,41,42^ The broad usage of HRRs to represent health regions facilitated substantial advances in our understanding of surgical health service delivery and informed quality improvement programs and policy initiatives. However, distinct limitations of the Dartmouth approach, including the age of data, considerable shifts in the health care landscape, and concerns regarding applicability to distinct conditions or patient populations, have prompted interest in network science approaches that use empirical data specific to the patient populations of interest.

Community detection methods have been applied to identify referral and transfer networks for complex cancer care, adult critical care, neonatal intensive care, stroke, and traumatic injury with distinct communities and patterns identified for each.^43,44,45,46,47^ When compared directly with the Dartmouth approach, MO has shown superior in ability to localize communities, with the advantage of being scale-flexible and automated, and therefore responsive to changes in the health care landscape over time or during episodes of disruption.^36^ Our work adds to this growing application of network science to health systems by identifying regional networks for nontraumatic surgical emergencies. These findings are important because EGS represents a high public health burden with a larger range of potential treatment facilities than other highly centralized conditions like complex cancer care or cardiovascular surgery. Our findings demonstrate regional networks and IHT patterns that are distinct from other conditions and emphasize the need to use contemporary, empirical data to identify care regions for health system planning.

National organizations including the AAST and ACS have responded to evidence detailing the burden of EGS with calls to improve health systems for nontraumatic surgical emergencies.^4,13^ In addition to the high volume of admissions, EGS accounts for 11% of all surgical procedures but more than 50% of all surgical mortality, and at over $28 billion per year, represent more than one-quarter of all inpatient costs.^1,2,3,48^ Furthermore, wide disparities exist in geographic access to emergency surgical care, with rural, racially and ethnically minoritized, and socioeconomically vulnerable patients most severely affected.^49,50^ The identification of EGS-specific regional networks is critical to efforts to design health systems that address this need. First, identifying communities of hospitals closely connected through catchment areas and existing care patterns provides a framework on which to develop policies and infrastructure that can expedite care for the sickest patients. Standardized triage and transfer pathways, centralized coordination centers for IHTs, and use of remote telemedicine consultation services have all been proposed as ways to improve care.^13,51^ Second, delineating EGS-specific networks may facilitate the development of regional quality collaboratives, which have been shown to improve outcomes through sharing benchmarking of outcomes, sharing best practices, implementing guidelines, and peer coaching to improve lower performing centers.^52,53^ In both cases, optimal implementation will require a data-informed understanding of the geographic regions and hospitals pertinent to each EGS network.

Finally, identifying geographic boundaries of EGS-specific health regions will strengthen our ability to monitor population-level access and outcomes and develop interventions to address inequities. As noted by the National Academy of Medicine in their 2013 report, *Towards Quality Measures for Population Health*, the term *population health* is often used narrowly to describe a “patient panel or group of covered lives,” and instead recommend the term *total population health* be used to refer to “the health of all persons living in a specified geopolitical area.”^54^ Carr and colleagues,^55^ in their assessment of emergency care regions, remark that while health system planning is endorsed by national and state entities for emergency care, outcomes are assessed at the facility level, so there is little incentive for hospitals to cooperate to improve regional care. A key example of this is the ACS Trauma Quality Improvement Program, where trauma systems include regional planning and cooperation; however, outcomes are assessed by each hospital for those admitted to their facility and fail to hold the regional system accountable for coordination between hospitals, transfer processes, or disparities within the region. As we move toward developing regionalized systems for EGS care, defining EGS care regions presents an opportunity to include monitoring of total population health and develop programs that encourage cooperation among elements of the system to address population-level inequities.

### Limitations

Limitations of this analysis include those inherent to the use of administrative claims data.^56^ While we used *ICD-10-CM* diagnosis codes for conditions identified by the AAST and ACS to be essential to EGS programs and services, the HCUP datasets do not include clinician identifiers, and thus we are unable to verify the involvement of a surgeon during the encounter. Our geographic precision was limited to the zip code tabulation area level, while small geographic units, such as census tracts or block groups, may be preferable in certain circumstances (eg, dense urban areas) for health system planning. Our identification of EGS communities was further limited by the use of single-state datasets, which cannot account for border-crossing behaviors. We tried to limit this by selecting states that are bordered on one side by water and have other natural boundaries (eg, mountain ranges or the US-Canada border) that are likely to discourage out-of-state movement. Despite these limitations, our findings that MO-detected EGS care regions performed better than the Dartmouth approach are strengthened by the consistency between 2 geographically distinct states.

## Conclusions

Network analysis methods to detect health regions specific to EGS care are poised to make unique contributions to the development of regionalized care systems for nontraumatic surgical emergencies. Compared with the long-standing Dartmouth HRRs, MO-detected EGS regions offer superior localization of care regions and hospital communities that reflect current utilization patterns and can be immediately applied to the development of regional policies, infrastructure, and quality collaboratives to improve care of this high-risk patient population.

## Notes

Supplement 1. **eTable.** Emergency General Surgery *ICD-10 CM* Diagnosis Codes

Supplement 2.
Data Sharing Statement
