# Supplementary material for: Application of Community Detection Methods to Identify Emergency General Surgery–Specific Regional Networks
Source: JAMA Netw Open. 2024 Oct 15;7(10):e2439509. doi: 10.1001/jamanetworkopen.2024.39509 (PMC11581592; doi:10.1001/jamanetworkopen.2024.39509)
Supplement: Supplement 1. — eTable. Emergency General Surgery ICD-10 CM Diagnosis Codes [file jamanetwopen-e2439509-s001.pdf]

Supplemental Online Content

Han J, Wan N, Horns JJ, McCrum ML. Application of community detection methods to identify emergency general surgery–specific regional networks. *JAMA Netw. Open*. 2024;7(10):e2439509. doi:10.1001/jamanetworkopen.2024.39509

eTable. Emergency General Surgery *ICD-10 CM* Diagnosis Codes

This supplemental material has been provided by the authors to give readers additional information about their work.

© 2024 Han J et al. *JAMA Network Open*.

eTable. Emergency General Surgery *ICD-10 CM* Diagnosis Codes

**EGS Condition**	**ICD-10 CM Diagnosis Codes***
Appendicitis	K35, K358, K3580, K3589, K35890, K35891, K37, K352, K3520, K3520, K3521, K353, K3530, K3531, K3532, K3533
Cholecystitis	K8000, K8001, K8012, K8013, K8042, K8043, K8046, K8047, K8062, K0863, K8066, K8067, K810, K812, K820, K821, K822, K823
Diverticulitis	K5732, K5733, K5720, K5721
Esophageal Perforation	K2210, K2211, K226, K223, S27813A
Perforated Ulcer	K251, K252, K255, K256, K261, K262, K265, K266, K271, K272, K275, K276, K659, K651, K6811, K6812, K6819, K650, N733, N735
Intestinal Obstruction	K561, K562, K563, K5641, K5649, K565, K5660, K5669, K913, K55021, K55022, K55029, K55041, K55042, K55049, K55061, K55062, K55069, K659, K631, K651, K650, N733, N735<br>With perforation: K631, K651, K650, K659, N733, N735
Infectious Colitis	A0471, A0472, K55031, K55032, K55039, K55041, K55049, K55051, K55052, K55059, K55061, K55069, K559, K631, K650, K651, K659, N733, N735, K55042, K55062
Intestinal Ischemia	K55011, K55012, K55019, K55031, K55032, K55039, K55051, K55052, K55059, K559, K55021, K55029, K55041, K55049, K55061, K55069<br>K55021, K55029, K55041, K55049, K55061, K55069<br>With perforation: K631, K651, K650, K659, N733, N735
Acute Pancreatitis	K8500, K8510, K8520, K8530, K8580, K8590, K8501, K8511, K8521, K8531, K8581, K8591, K8502, K8512, K8522, K8532, K8582, K8592, K6811, K6812, K6819, K529, K55011, K55012, K55019, K55021, K55022, K55029, K55031, K55032, K55039, K55041, K55042, K55049, K55051, K55052, K55059, K55061, K55062, K55069, K559, K631, K650, K651, K659, N733, N735
Perirectal abscess	K610, K611, K612, K613, K614, N493, N7689, A480, I96, M726
Hernia	K4020, K4021, K4090, K4091, K4120, K4121, K4190, K4191, K429, K432, K435, K439, K449, K458, K469, K4000, K4001, K4030, K4031, K4100 K4101, K4130, K4131, K420, K430, K433, K436, K440, K450, K460, K4010, K4011, K4040, K4041,

© 2024 Han J et al. *JAMA Network Open*.

	K4110, K4111, K4140, K4141, K421, K431, K434, K437, K441, K451, K461<br>With perforation: K631, K651, K650, K659, N733, N735
Soft Tissue Infection	A46, I891, L00, L0100, L0101, L0102, L0103, L0109, L011, L03011<br>L03012, L03019, L03021, L03022, L03029, L03031<br>L03032, L03039, L03041, L03042, L03049, L03111<br>L03112, L03113, L03114, L03115, L03116, L03119<br>L03121, L03122, L03123, L03124, L03125, L03126<br>L03129, L03211, L03212, L03221, L03222, L03311<br>L03312, L03313, L03314, L03315, L03316, L03317<br>L03319, L03321, L03322, L03323, L03324, L03325, L03326, L03327, L03329, L03811, L03818, L03891, L03898, L0390, L0391, L0882, L0889, L089, L0201, L0202, L0203, L0211, L0212, L0213, L02211, L02212, L02213, L02214, L02215, L02216, L02219, L02221, L02222, L02223, L02224, L02225, L02226, L02229, L02231, L02232, L02233, L02234, L02235, L02236, L02239, L0231, L0232, L0233, L02411, L02412, L02413, L02414, L02415, L02416, L02419, L02421, L02422, L02423, L02424, L02425, L02426, L02429, L02431, L02432, L02433, L02434, L02435, L02436, L02439, L02511, L02512, L02519, L02521, L02522, L02529, L02531, L02532, L02539, L02611, L02612, L02619, L02621, L02622, L02629, L02631, L02632, L02639, L02811, L02818, L02821, L02828, L02831, L02838, L0291, L0292, L0293, L0501, L0502, L080, M60000, M60001, M60002, M60003, M60004, M60005, M60009, M60011, M60012, M60019, M60021, M60022, M60029, M60031, M60032, M60039, M60041, M60042, M60043, M60044, M60045, M60046, M60051, M60052, M60059, M60061, M60062, M60069, M60070, M60071, M60072, M60073, M60074, M60075, M60076, M60077, M60078, M6008, M6009, M6500, M65011, M65012, M65019, M65021, M65022, M65029, M65031, M65032, M65039, M65041, M65042, M65049, M65051, M65052, M65059, M65061, M65062, M65069, M65071, M65072, M65079, M6508, M6510, M65111, M65112, M65119, M65121, M65122, M65129, M65131, M65132, M65139, M65141, M65142, M65149, M65151, M65152, M65159, M65161, M65162, M65169, M65171, M65172, M65179, M6518, M6519, M728, M726, N493,

© 2024 Han J et al. *JAMA Network Open*.

	A480, E0852, E0952, E1052, E1152, E1352, I70261, I70262, I70263, I70268, I70269, I70361, I70362, I70363, I70368, I70369, I70461, I70462, I70463, I70468, I70469, I70561, I70562, I70563, I70568, I70569, I70661, I70662, I70663, I70668, I70669, I70761, I70762, I70763, I70768, I70769, I96
Intestinal Perforation with Peritonitis (May be applied to multiple conditions as per Scott et al, 2021)	K631, K651, K650, K659, N733, N735
Pain Intestinal Infarction (May be applied to multiple conditions as per Scott et al, 2021)	K55022, K55042, K55062

* Mapping logic followed that presented in: Scott JW, Staudenmayer K, Sangji N, Fan Z, Hemmila M, Utter G. Evaluating the association between American Association for the Surgery of Trauma emergency general surgery anatomic severity grades and clinical outcomes using national claims data. *J Trauma Acute Care Surg*. 2021;90(2):296-304.

© 2024 Han J et al. *JAMA Network Open*.
