# Supplementary material for: Application of Community Detection Methods to Identify Emergency General Surgery–Specific Regional Networks
Source: JAMA Netw Open. 2024 Oct 15;7(10):e2439509. doi: 10.1001/jamanetworkopen.2024.39509 (PMC11581592; doi:10.1001/jamanetworkopen.2024.39509)
Supplement: Supplement 2. — Data Sharing Statement [file jamanetwopen-e2439509-s002.pdf]

Data Sharing Statement

Han. Application of Community Detection Methods to Identify Emergency General Surgery–Specific Regional Networks. *JAMA Netw Open*. Published October 15, 2024. doi:10.1001/jamanetworkopen.2024.39509

Data
Data available: Yes
Data types: Deidentified participant data
How to access data: Available upon request: marta.mccrum@hsc.utah.edu
When available: With publication

Supporting Documents
Document types: Statistical/analytic code
How to access documents: Available upon request: marta.mccrum@hsc.utah.edu
When available: With publication

Additional Information
Who can access the data: Researchers who proposed use of the data has been approved
Types of analyses: Approved research purposes
Mechanisms of data availability: After approval of proposal, with signed data access agreement
